# Complete mitochondrial genome of the medicinal fungus *Ophiocordyceps sinensis*

**DOI:** 10.1038/srep13892

**Published:** 2015-09-15

**Authors:** Yi Li, Xiao-Di Hu, Rui-Heng Yang, Tom Hsiang, Ke Wang, De-Quan Liang, Fan Liang, De-Ming Cao, Fan Zhou, Ge Wen, Yi-Jian Yao

**Affiliations:** 1 State Key Laboratory of Mycology, Institute of Microbiology, Chinese Academy of Sciences, Beijing 100101, China; 2 College of Plant Protection, Fujian Agriculture and Forestry University, Fuzhou 350002, China; 3 University of Chinese Academy of Sciences, Beijing 100049, China; 4 School of Environmental Sciences, University of Guelph, Ontario, N1G 2W1, Canada; 5 Nextomics Biosciences Co., Ltd., Wuhan 430075, China

## Abstract

As part of a genome sequencing project for *Ophiocordyceps sinensis*, strain 1229, a complete mitochondrial (mt) genome was assembled as a single circular dsDNA of 157,510 bp, one of the largest reported for fungi. Conserved genes including the large and small rRNA subunits, 27 tRNA and 15 protein-coding genes, were identified. In addition, 58 non-conserved open reading frames (ncORFs) in the intergenic and intronic regions were also identified. Transcription analyses using RNA-Seq validated the expression of most conserved genes and ncORFs. Fifty-two introns (groups I and II) were found within conserved genes, accounting for 68.5% of the genome. Thirty-two homing endonucleases (HEs) with motif patterns LAGLIDADG (21) and GIY-YIG (11) were identified in group I introns. The ncORFs found in group II introns mostly encoded reverse transcriptases (RTs). As in other hypocrealean fungi, gene contents and order were found to be conserved in the mt genome of *O. sinensis*, but the genome size was enlarged by longer intergenic regions and numerous introns. Intergenic and intronic regions were composed of abundant repetitive sequences usually associated with mobile elements. It is likely that intronic ncORFs, which encode RTs and HEs, may have contributed to the enlarged mt genome of *O. sinensis*.

*Ophiocordyceps sinensis* (Berk.) G.H. Sung, J.M. Sung, Hywel-Jones & Spatafora, placed systematically as *Ophiocordycipitaceae*, *Hypocreales*, *Hypocreomycetidae*, *Sordariomycetes*, *Ascomycota*, is a fungal pathogen that parasitizes larvae of Himalayan ghost moths in the *Hepialidae*^1^. It is distributed on the Tibetan Plateau and surrounding high elevation regions, including Tibet, Gansu, Qinghai, Sichuan and Yunnan provinces in China and certain areas of the southern Himalayas in Bhutan, India and Nepal^2^. This fungus has been used as a traditional medicine in China for centuries^3^. Due to its host specificity, confined distribution and overexploitation in the past decades, annual yield of *O. sinensis* has decreased and therefore this fungus been listed as an endangered species under the Chinese Second Class of State Protection^4^. Naturally produced *O. sinensis* is by weight worth more than gold, or even reaching four times as much, especially for product of high quality as represented by superior aesthetics.

Because of its economic value, *O*. *sinensis* has gained increasing scientific attention in recent decades. Genetic diversity was investigated for this fungus and its host insects using multigene approaches^5^,^6^,^7^. Both mating type genes (*MAT1–1*/*MAT1–2*) were found to occur within the same isolate and expressed under vegetative conditions, suggesting a capability for self-fertility in the species^8^. Genome sequencing confirmed this homothallism in *O. sinensis*, and revealed the repeat-driven genome expansion of this fungus^9^. In addition, transcriptome analysis demonstrated the expression of both mating type genes in fresh fruiting bodies^10^. Although the nuclear genome and transcriptome have been published, the mitochondrial (mt) genome has not yet been reported.

Mitochondria are cellular organelles which play various essential roles in eukaryotic cells. In addition to the primary function in respiratory metabolism and energy production, mitochondria are also involved in many other processes such as calcium homeostasis, cell aging and apoptosis^11^. An endosymbiotic hypothesis suggests that the ancestor of mitochondria was most closely related to *Alphaproteobacteria*^12^. Gene loss and organization changes of the mt genome have occurred during the evolutionary process of the endosymbiont becoming a cellular organelle^13^. Previous studies indicated that the loss of ancestral bacterial genes resulted in small and compact mt genomes^14^, especially within fungi^15^.

Fungal mt genomes are single circular dsDNA molecules in most cases and generally encode 14 essential genes required for electron transport and oxidative phosphorylation (*atp6*,*8*,*9*; *cob*, *cox1–3*, *nad1–6 and nad4L*), small (*rns*) and large (*rnl*) subunit mitochondrial rRNAs and a set of tRNA genes^15^. Genes are typically encoded on the same sense mtDNA strand in most ascomycetes, while encoded on either mtDNA strand in basidiomycetes^16^. Although gene contents are almost always conserved, mt genome sizes and gene synteny are highly variable. The mt genome size in higher fungi (*Ascomycota* and *Basidiomycota*) varies among species and is known to range from 18,844 bp for *Hanseniaspora uvarum* in *Saccharomycetales*^17^ to 235,849 bp for basidiomycetous *Rhizoctonia praticola*^18^. The variation of mt genome size can be explained by variations in the length and organization of intergenic regions, or differences in the number and length of introns^19^. For instance, 80% of the 156 kb of *Phlebia radiata* mt genome was composed of intronic and intergenic regions^20^, while no introns was observed in the 49.7 kb *Schizophyllum commune* mt genome^21^. Gene order variation could be due to repetitive DNA in the form of introns with self-splicing and insertion endonuclease activity, the introduction of new genes through horizontal gene transfer (HGT), or the distribution of transfer RNAs (tRNAs) that display editing, excision and integration capabilities^16^.

In this study, the mt genome of *O. sinensis*, strain 1229, was sequenced using third generation sequencing technology on a PacBio RS II sequencing platform, annotated and compared with other fungal mt genomes. In particular, detailed comparisons with known mt genomes of hypocrealean fungi were made and analyzed. Possible reasons for the enlarged mt genome of *O. sinensis* are also discussed.

## Results

### DNA and RNA extraction

Genomic DNA was extracted from mycelia produced in liquid culture (usually 10 μg genomic DNA from 500 mg dried mycelium) and sent for sequencing on a PacBio Platform. Approximately 150 μg of total RNA was isolated from 1 g frozen mycelium and applied to Illumina HiSeq^TM^ 2500.

### Conserved genes in the mt genome of *Ophiocordyceps sinensis*

**Figure 1:**
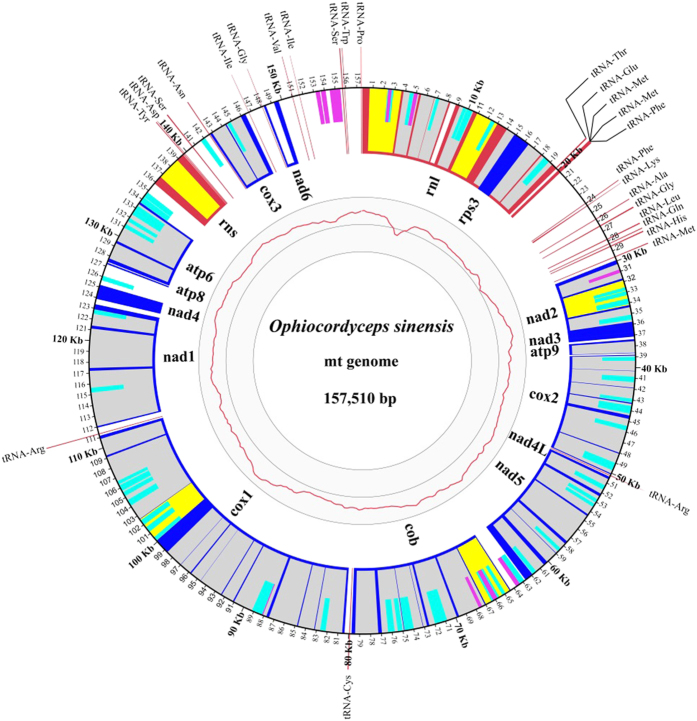
Genetic map of the mitochondrial genome of *Ophiocordyceps sinensis*. Dark blue radial lines or blocks indicate exons of conserved protein-coding genes, with gene names labelled on the inner circle; red boxes indicate the *rnl* and *rns* genes with the *rps3* nested within an intron of the *rnl* gene; thin red lines protruding outside of the outer circle indicate tRNAs. Introns are shown in shading as group I (grey), group II (yellow) or unclassified (white). Intronic and, less frequently, intergenic predicted ORFs (ncORFs > 300 bp) were shown as half-height bars representing sense strand of ncORFs (light blue) or anti-sense strand of ncORFs (magenta). The initiation codon of *rnl* gene was set as the start of mtDNA. Three fine inner circles represented sequence coverage with the outermost at 800× and the innermost at 0×.

A total of 13,751 reads (85,024,932 bp) were identified as mitochondrial among 179,974 reads (1,453,005,112 bp) of the raw sequencing output for the whole genome of *O. sinensis* (The analyses of the genome will be reported in a separate paper). The lengths of the putative mitochondrial reads ranged from 502 bp to 21,094 bp with an average length of 6,904 bp, reaching a coverage depth of 565 over the mt genome of the species. The mitochondrial reads were passed through the program BLASR and assembled with Celera Assembler program and Quiver, resulting in a circular DNA of 157,510 bp (Fig. 1).

The mt genome of *O. sinensis* had a low GC content of 30.2% and contained a set of 14 protein-coding genes conserved among fungi (Supplementary Table S2), including seven subunits of the electron transport complex I (*nad1*, *nad2*, *nad3*, *nad4*, *nad4L*, *nad5* and *nad6*), one subunit of complex III (*cob*), three subunits of complex IV (*cox1*, *cox2* and *cox3*) and three F0 subunits of the ATP-synthase complex (*atp6*, *atp8* and *atp9*). The *rps3* gene which encodes 40S ribosomal protein S3 was identified within an intron of *rnl*, as is the case with most filamentous ascomycetes^22^. In addition to these 15 protein-coding genes, 27 tRNA genes and genes for the large and small ribosomal RNA (*rnl* and *rns*) were also identified in the *O. sinensis* mt genome. All conserved protein coding and RNA genes (tRNA, rRNA) were found on the positive strand and oriented clockwise. As found in mt genomes of *Rhizoctonia solani*^18^ and *Pleurotus ostreatus*^23^, the *O. sinensis nad2*/*nad3* genes and the *nad4L*/*nad5* genes were respectively joined and fused (Fig. 1). The ATG initiation codon of the *nad3* gene immediately followed the TAA termination codon of the *nad2* gene and the termination codon of *nad4L* (TAA) uses the same nucleotide A with the initiation codon (ATG) of *nad5* (Fig. 1). For all the other genes, either long or short intergenic regions were present (Fig. 1).

### Transfer RNAs

A total of 27 tRNA genes were found in the mt genome of *O. sinensis* corresponding to 20 amino acids (Supplementary Table S3). As shown in Supplementary Table S3, three tRNA genes with different sequences and same anticodon (CAU) were found for tRNA-Met and two each were found for tRNA-Arg, tRNA-Gly, tRNA-Ile, tRNA-Leu and tRNA-Ser. Among these five tRNAs with two coding genes, tRNA-Gly genes had the same anticodon from different sequences, while sequences and anticodons differed for each of the other four tRNAs. For the remaining 14 tRNAs, only one gene each was found (Table S3). Similar to many mt genomes of the class *Sordariomycetes*^16^, tRNA genes of *O. sinensis* mt genome were found to be clustered. The main cluster consisted of 24 tRNA genes confined to a 47 kb area around the *rnl* gene, with three tRNA genes (tRNA-Arg, tRNA-Cys and tRNA-Arg) dispersed across the mt genome (Fig. 1). The presence of tRNA-Trp recognizing the UGA codon suggested that *O. sinensis* mt genome may follow the Mold, Protozoan, and Coelenterate Mitochondrial Code rather than the standard code.

### Intergenic and intronic regions in the mt genome of *Ophiocodyceps sinensis*

**Figure 2:**
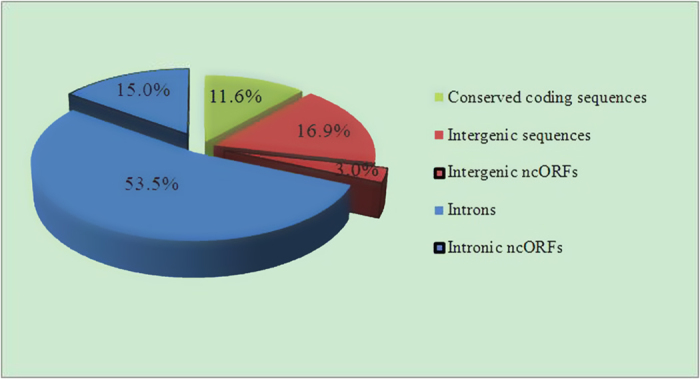
Composition of the *Ophiocordyceps sinensis* mt genome showing proportion of coding, intergenic, intronic regions and ncORFs. Conserved coding sequences refer to the conserved protein-coding genes, rRNA and tRNA genes. ncORFs refer to hypothetical protein-coding sequences longer than 300 bp identified by ORF Finder.

The *O. sinensis* mtDNA is the third largest (157,510 bp) among 176 fungal mt genomes published to date (16 April 2015), following only *Rhizoctonia solani* (235,849 bp) and *Sclerotinia borealis* (203,051 bp). Exons of 15 protein-coding, 2 rRNA and 27 tRNA genes covered 13.9% (21,863 bp) of the mt genome. The intergenic sequences had a total length of 31,382 bp covering 19.9% (Fig. 2) of the genome. Accounting for 68.5% (107,611 bp) of the genome (Fig. 2), 52 introns were detected by RNAweasel combined with a manual approach using ClustalX.

A total of 52 introns were found in 11 of the 15 protein-coding and 2 rRNA genes (*rnl* and *rns*). The sizes of the introns ranged from 522 bp (intron 5 in *rnl*) to 6435 bp (intron 11 in *cox1*), with an average length of about 2000 bp (Supplementary Table S4). Most introns were classified into group I (44 out of 52) and only six in group II (Table S4). Two short introns of 522 bp (intron 5 in *rnl*) or 777 bp (intron of *nad6*) did not belong to either group (Fig. 1). Within group I, the introns were further classified into subgroups IA (8), IB (18), IC1 (2), IC2 (12) and ID (4) (Table S4). Different groups of introns were found within a single gene, e.g. 11 IB, one ID and one group II introns in *cox1* gene (Table S4).

In the *cox1* gene, 12 group I and one group II introns were found accounting for 94.9% of the open reading frame (ORF). The *rnl* gene had six group I, two group II and one uncertain introns representing 81.6% of the whole ORF. The *cob* gene had five group I and one group II introns (92.5%). The *nad2* gene had two group I and one group II introns (76.7%). The *rns* gene had one group II intron (55.6%). The *nad6* gene had one unclassified intron (53.8%). For the remaining seven protein-coding genes that contained only group I introns, *cox2*, *nad5* and *nad1* possessed five, five and three introns representing 93.8%, 81.5% and 89.8% of the ORFs respectively; *atp6* and *cox3* possessed two introns each representing 87.5% and 78.2%, respectively; and *atp9* and *nad4L* each possessed a single intron representing 82.6% and 84.8%, respectively.

### Predicted non-conserved open reading frames (ncORFs) in the intergenic and intronic regions

ORF Finder identified 58 ncORFs longer than 300 bp in the intergenic and intronic regions, accounting for 18.0% of the mt genome (Fig. 2). Among them, 9 were present in intergenic regions, 11 in group II introns and the remaining 38 in Group I introns (Supplementary Table S5). All of the ncORFs in group II introns were found to encode reverse transcriptases (RTs), except ncORF39 which showed no homology with any protein. Most of group I intronic ncORFs were associated with homing endonucleases (HEs) with motif patterns LAGLIDADG (21) or GIY-YIG (11). One ncORF in intron 5 (group I) of the *cob* gene showed possible similarity to reverse gyrase (e-value = 0.00322). The other five ncORFs within group I introns showed no significant similarity to any known proteins, and were defined as hypothetical. ncORFs in intergenic regions were found to encode proteins with more variegated function, including fibronectin-attachment protein (ncORF26), DNase SDA1 (ncORF27) and DNA-dependent RNA polymerase (ncORF56 and ncORF57). Most ncORFs, 48 out of 58, were located in the sense strand while ncORFs located in anti-sense strand encoded similar types of proteins (i.e. HEs and RTs). Among these 10 ncORFs on the anti-sense strand, two (ncORF2 and ncORF30) were observed inside other ncORFs (ncORF1 and ncORF29) encoding the same RTs as on the sense strand.

### Repetitive sequences in the mt genome of *Ophiocordyceps sinensis*

**Figure 3:**
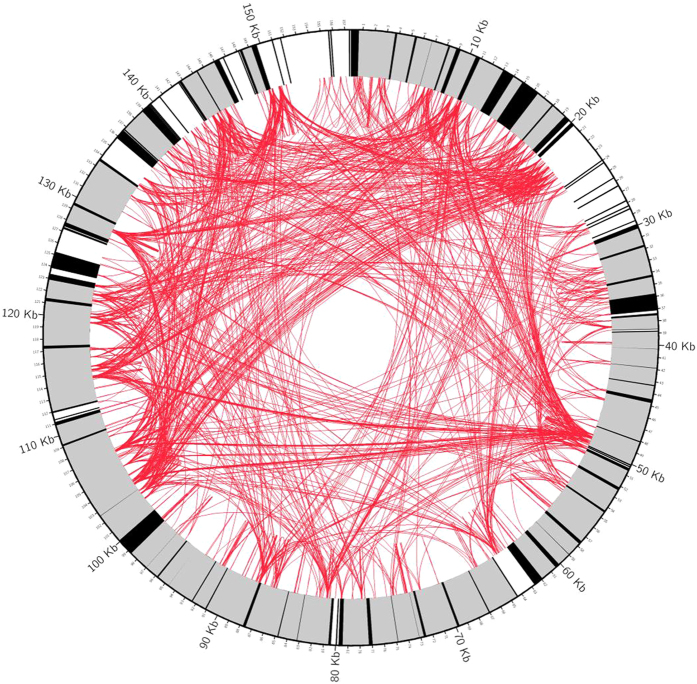
Dispersed and inverted repeat sequences in the mt genome of *Ophiocordyceps sinensis*. Red ribbons connect regions of significant (e-value < 10^−5^) nucleotide sequence similarity. Black bars in the outer ring represent conserved protein-coding regions, and rRNA and tRNA genes; gray bars are for introns; and white bars are for intergenic regions.

A local self BLASTn of the 157,510 bp mt genome against itself revealed 1251 repetitive sequences with a total length of 108,503 bp, accounting for 69.0% of the *O. sinensis* mt genome. The size of repeats ranged from 28 bp to 863 bp. The TRF program found 45 tandem repeats, totalling 2826 bp and accounting for 1.8% of the genome, ranging from 2 to 123 bp in size. Total length of simple sequence repeats (SSRs) identified by MISA was 848 bp (<0.6% of the mt genome). REPuter was used to identify 31 forward (in total 5092 bp), five reverse (684 bp) and 14 palindromic (2030 bp) repeats, accounting for 5.0% of the mt genome. No complement repeat was identified by REPuter. EMBOSS identified 243 short inverted repeats with the largest size at 24 bp, accounting for 1.8% (2816 bp) of the genome. The most abundant repeat types were dispersed and inverted repeat sequences. The vast majority of repeats of various types (direct, reverse, inverted, SSRs and tandem repeats) were located in the intergenic and intronic regions, with the intronic regions being the most frequent (as shown in Fig. 3 for dispersed and inverted repeats, SSRs and tandem repeats were analysed by their location separately). Interestingly, the region from 24 to 102 kb, except for a few hotspots, showed less frequent repeated sequences (Fig. 3).

### Phylogenetic analyses and gene order in mt genomes of *Hypocreales*

**Figure 4:**
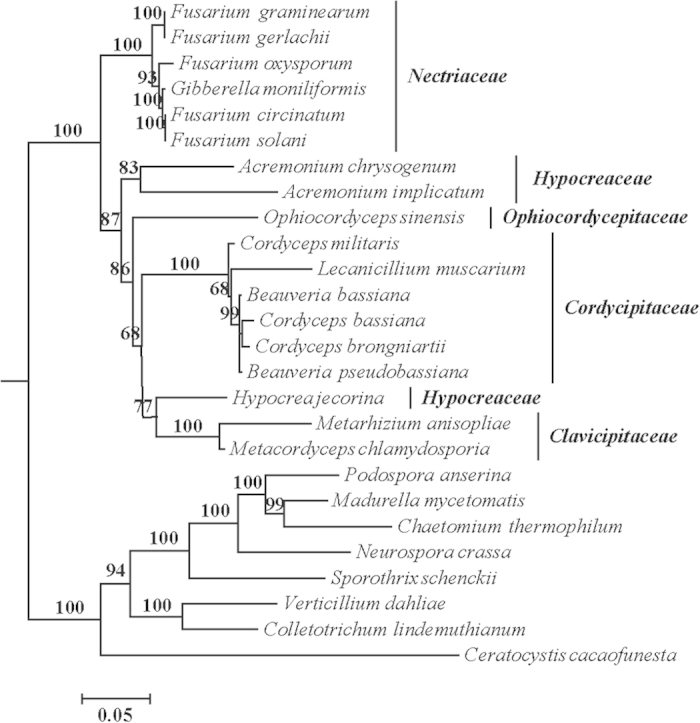
Phylogenetic relationships among 18 taxa of *Hypocreales* based on mt protein sequences of 14 conserved protein-coding genes (i.e. *cox1*, *cox2*, *cox3*, *atp6*, *atp8*, *atp9*, *nad1*, *nad2*, *nad3*, *nad4*, *nad5*, *nad4L* and *nad6*). Bootstap values were shown above the nodes. Other orders in the class *Sordariomycetes* were used as outgroups.

Phylogenetic analyses were performed using 3808 aa sequences of 14 mt protein-coding genes from 26 taxa. The results of Maximum Likelihood (ML) analyses revealed *Hypocreales* as a monophyletic group forming a well-supported clade (BP = 100%). Within the hypocrealean clade, four families can be recognized by very strongly supported subclades or only represented by a single taxon, i.e. *Nectriaceae* (BP = 100%), *Ophiocordycipitaceae* (single taxon), *Cordycipitaceae* (BP = 100%) and *Clavicipitaceae* (BP = 100%) (Fig. 4). The family *Hypocreaceae* appeared to be polyphyletic, as one of the three species of the family included in this analysis, *Hypocrea jecorina*, was placed as a sister to the *Clavicipitaceae* subclade with 77% BP support (Fig. 4).

The contents and order of conserved mt genes were consistent among the species of *Hypocreales* except the two *Acremonium* species (members of *Hypocreaceae*), in which several genes were lost (*rps3* in *A*. *chrysogenum*; *cob*, *cox3* and *nad6* in *A*. *implicatum*) or changed in order (*nad4* in *A*. *implicatum*) (Figure S1). In the outgroups, represented by other members of *Sordariomycetes*, gene contents and order were much more variable but the microascalean *Ceratocystis cacaofunesta* shared the same gene contents and order as the hypocrealean (Figure S1).

### Transcription analysis of conserved protein-coding genes and ncORFs

The expression of conserved protein-coding genes and predicted ncORFs was validated by RNA-Seq. After filtering, 46,748,662 reads totalling 4.63 Gb were retained. Among these, 301,977 reads were associated with mitochondria, and 164,909 reads were mapped to mt protein-coding and mt RNA genes (rRNAs and tRNAs) and 137,068 to mt ncORFs. Transcription analyses showed that conserved genes including 15 protein-coding genes, 2 rRNA and 20 out of 27 tRNA genes were expressed, and most of the predicted ncORFs were transcriptionally active, especially ncORFs encoded on the sense strand (Supplementary Table S6). Although most active ncORFs were on the sense strand, a DNase SDA1 anti-sense (ncORF27) on the anti-sense strand was highly expressed (RPKM > 600). ncORF2 and ncORF30 on the anti-sense strand were fully nested within their sense strand counterparts (ncORF1 and ncORF29, respectively), and all the four encode reverse transcriptases. However, the two anti-sense strand ncORFs had no detectable expression, while the larger sense strand ncORF counterparts were highly expressed (Supplementary Table S5). Low RPKM values indicated that DNA-dependent RNA polymerases encoded on the anti-sense strand (ncORF56 and ncORF56) in the intergenic region were not active under the growth conditions when RNA was extracted (Supplementary Table S6). Conserved genes with the highest RPKM were two rRNA genes (*rnl* and *rns*), while 10 ncORFs with the highest RPKM (upper quartile) were those encoding for reverse transcriptases and homing endonucleases (Supplementary Tables S5 and S6).

## Discussion

The 157,510 bp mt genome from *O. sinensis*, strain 1229 described here, is the third largest reported fungal mt genome and the second largest among ascomycetes. The alphaproteobacterial ancestor of mitochondria probably had a genome size greater than 1 Mb^12^. For example, a strain (IMCC9063) from the SAR11 clade of *Alphaproteobacteria* has a genome size of 1.28 Mb encoding 1447 proteins^24^. It is speculated that mt genomes were highly reduced (by 10–1000-fold) in protein gene contents in descent from their alphaproteobacterial ancestor, retaining genes almost exclusively involved in respiration and protein synthesis^13^. Although the gene contents of mitochondria were largely conserved, typical fungal mt genomes usually encode 30–40 genes^15^. However, their sizes vary greatly, from 12 kb of a mycoparasitic species *Rozella allomycis* in *Cryptomycota* to over 235 kb for *Rhizoctonia solani* in *Basidiomycota*^25^. Fungal species with enlarged mt genomes usually involve members of *Basidiomycota* subphylum *Agaricomycotina*, e.g. *Agaricus bisporus*^26^ (135 kb), *Phlebia radiata*^20^ (156 kb) and *Rhizoctonia solani*^18^ (235 kb), but this also has been found with a few filamentous ascomycetes, e.g. *Podospora anserina*^27^ (over 100 kb) and *Chaetomium thermophilum var. thermophilum*^28^ (over 127 kb).

Species in *Hypocreales*, to which *O. sinensis* belongs, generally have similar mt genome sizes, i.e. 24,673 bp in *Metarhizium anisopliae*, 25,615 bp in *Metacordyceps chlamydosporia*, 28,006 bp in *Beauveria pseudobassiana*, 29,961 bp in *Beauveria bassiana* and 33,277 bp in *Cordyceps militaris*^25^ (Figure S1). However, *O. sinensis* has an unusually enlarged mt genome size (157,510 bp). The size of the nuclear genome of this fungus is also expanded, estimated at over 120 Mb^9^, which is larger than most other *Ascomycota*. As in another expanded fungal genome (*Tuber melanosporum*, 125 Mb)^29^, the expanded nuclear genome could be due to a large proportion of transposable elements^9^, which are mobile elements often resulting in duplications (repeats). However, transposable elements have not been commonly reported in fungal mt genomes, other processes might be involved in the expansion of mt genome.

It has been reported that size variation of mt genome may be caused by the length and organization of intergenic regions or the presence of introns (group I and II) of various size^19^. Intergenic and intronic sequences in the mt genome of *O. sinensis* were found to have a total length of 31,382 bp and 107,859 bp each, contributing 19.9% and 68.5% to the mt genome size, respectively (Fig. 2). Even if all the ncORFs were excluded, the intergenic and intronic sequences still accounted for 70.4% of the genome (Fig. 2). Similar situations have been reported in other fungal species with expanded mt genome, e.g. intronic and intergenic regions summing up to 80% of the 156 kb mtDNA sequence from *Phlebia radiata*, a basidiomycetous white-rot fungus^20^ and some 61 introns accounting a total of 125,394 bp in the second largest mt genome (203,051 bp) in the ascomycetous *Sclerotinia borealis*^30^. In the mt genome of *O. sinensis*, most intergenic and intronic regions were filled with repetitive sequences, with very few observed in coding regions (Fig. 3). Different repetitive sequences (direct, reverse, inverted, SSRs and tandem repeats), interspersed in the whole genome but more densely distributed from 102 to 24 kb clockwise (Fig. 3), accounted for more than 70% of the mt genome size. Various mobile elements in fungal mt genomes, e.g. LAGLIDADG and GIY-YIG homing endonucleases in group I introns and reverse transcriptases in group II introns^31^, were also found in *O. sinensis* (Table S5). Some of the repetitive sequences have been suggested to be mobile^32^. However, the true relationship between repetitive sequences leading to mt genome expansion and mobile elements requires further study.

Mitochondrial introns can be classified into two groups (groups I and II) according to their distinct and conserved RNA secondary structures^31^. Group I introns are further divided into subgroups (IA, IA3, IB, IC1, IC2, ID) based on phylogenetic analyses^31^. In general, group I introns are dominant in fungal mitochondrial genes with greater association for genes, e.g. *cox1*, *cob* and *rnl*, while group II introns are predominant in plant mt genomes^31^. In the mt genome of *O. sinensis*, 44 group I and 6 group II introns were identified in 12 protein-coding genes, except two unclassified short introns (intron of *nad6* and intron 5 in *rnl*). Within group I, the occurrence of subgroup introns were also uneven, subgroups IB and IC2 were apparently more frequent than subgroups IA, ID and IC1 (Table S4), in accordance with a previous report on mitochondrial introns^31^.

Although introns are often seen in mt genomes, the origin and modes of transmission of mitochondrial introns remain controversial. One hypothesis indicated that introns were abundant in the ancestral mt genes, but had subsequently been lost in most lineages^33^. While in angiosperms, mitochondrial introns can be acquired through horizontal gene transfer^34^. Both loss and gain events are required to explain the uneven distribution and evolutionary dynamics of mitochondrial introns^26^. Compared with other species in *Hypocreales* with smaller mt genomes such as *C. militaris*, *O. sinensis* has a mt genome with accumulated introns of various lengths (Table S4). These intronic sequences might be preserved from the ancestors or gained from other sources. Most mitochondrial group I introns in *O. sinensis* carried LAGLIDADG or GIY-YIG homing endonuclease genes, while group II introns have reverse transcriptase genes. Both of these groups of genes have been reported to facilitate the movement of introns into previously intronless genes or certain regions^31^, resulting in expansion of the mt genome size.

It is interesting to see the consistency of the gene contents and order of mt genomes from nearly all hypocrealean species (Figure S1). Hypocrealean fungi usually contain a whole set of coding genes conserved in their mt genomes, including *rnl*, *rps3* (usually located within a group I intron of *rnl* in hypocrealean fungi), *nad2*, *nad3*, *atp9*, cox2, *nad4L*, *nad5*, *cob*, *cox1*, *nad1*, *nad4*, *atp8*, *atp6*, *rns*, *cox3* and *nad6* (arranged clockwise), while in the exceptions, *A*. *chrysogenum* and *A*. *implicatum*, *rps3* was lost in the former, and *cob*, *cox3* and *nad6* were lost or not detected in the latter (Figure S1). The mitochondrial gene contents and order are highly variable among fungi but tend to be conserved in closely related fungal groups as described in a recent report^16^, in which six species of *Hypocreales* were included. In the present study, the exceptions in gene contents and order were revealed through an extended sampling coverage including a total of 18 hypocrealean species (Figure S1). However, if extended to *Sordariomycetes* (Figure S1) or to all fungi^16^,^21^, both gene contents and order vary greatly. For example, five genes, i.e. *rnl*, *cox2*, *cob*, *rns*, *cox3*, were lost in *Chaetomium thermophilum* and *atp9* was not detected in *Podospora anserina*^27^ (Figure S1). Furthermore, seven genes encoding subunits of the nicotinamide adenine dinucleotide dehydrogenase complex (*nad1*–*6*, *nad4L*) are present in most of the fungal mt genomes, but absent in three fission^14^ and several budding^35^ yeasts. The presence and absence of *rps3* are noted in different fungal lineages^13^. In addition, both tRNA distribution and repetitive sequences were reported to facilitate gene order variation^16^. tRNAs contribute to gene order variation as they themselves can change location^36^. tRNAs in the mt genome of *O. sinensis* were clustered into several locations (Fig. 1), showing a similar distribution pattern to other hypocrealean fungi. Repeats can favor recombination events, thereby promoting rearrangements that change gene order^37^,^38^. Simple and tandem repeats, especially those present in intergenic regions showed the strongest correlation with gene order^16^. Intronic ncORFs, particularly those encoding HEs, have a potential to insert copies in different locations within the genome, changing gene order, but strong correlations between gene order and the HEs genes have not been observed in a comparative analysis^16^. RTs, as one kind of transposable elements, also have the potential to move and thus change gene order. In this study, although abundant intronic ncORFs encoding HEs and RTs were identified in *O. sinensis*, gene order was not observed to be different from other hypocrealean fungi, possibly indicating a strong selective constraint on coding regions, as has been speculated^16^.

RNA-Seq analyses were designed mainly to investigate the transcriptional status of conserved genes and predicted ncORFs of the mt genome of *O*. *sinensis*. All of the 15 conserved protein-coding genes, 2 rRNA and 20 out of 27 tRNA genes were expressed, and most of the predicted ncORFs, especially those encoded on the sense strand, were transcriptionally active (Supplementary Tables S5 and S6). It is interesting to see the ncORFs encoding for RTs and HEs were highly transcribed (Supplementary Tables S5 and S6). Both RTs and HEs has been reported to have the ability to move around the genome and to increase the number of copies, resulting in sequence repeating^31^. Among the 49 intronic ncORFs, 43 were found to encode RTs and HEs. These mobile elements together with the surrounding sequences repeat extensively and occupy many parts of the genome (Fig. 3). The high activity of RTs and HEs, as detected by RNA-Seq analyses, may be responsible for the mt genome expansion, a significant biological feature possibly resulted from the adaption of high altitude of the Tibetan Plateau and also distinguishing the species from other hypocrealeans. The high expression of the rRNA genes may be in part due to sequence over-abundance^18^, a common character of mitochondria indicating a high incidence of protein synthesis.

Gene sequences of the mitochondrial DNA could be valuable for phylogenetic and diversity analyses because of their higher mutation rate than that of nuclear genes^15^. Phylogenetic analyses based on complete mt genomes have been performed for various organisms, especially insects^39^. In the present study, the phylogenetic analyses of *Hypocreales* using 14 protein-coding genes produced a similar backbone structure of the phylogenetic tree recognizing five families within the order *Hypocreales*, similar to that based on five nuclear genes^40^, although the family *Hypocreacea* became polyphyletic in the mt gene tree because of the separation of *Hypocrea jecorina* from other two species of the same family, *Acremonium chrysogenum* and *A. implicatum* (Fig. 4). Significant differences have also been found in the gene contents and order of mt genomes supporting the separation of *H*. *jecorina* from the other two species of the class *Hypocreacea* (Figure S1). Some of the members of *Hypocreales* have been included in phylogenetic analyses using protein-coding genes found in fungal mt genomes in previous work^16^,^30^, however the analyses presented here are the most comprehensive based on the available mt genome data from *Sordariomycetes*, including 18 species of *Hypocreales*.

## Methods

### Fungal cultivation

The strain 1229 of *O. sinensis* was isolated from a single ascospore of a mature specimen collected from Guoluo, Qinghai Province, China. The stock was maintained on wheat bran plates (Potato Dextrose Agar supplemented with 5 g/l wheat bran and 0.5 g/l peptone) at 10 °C. Seed cultures were grown in 250-ml Erlenmeyer flasks, containing 50 ml wheat bran liquid culture medium, shaking 100 rpm at 18 °C for 15 d. The seed cultures (5 ml) were transferred to 250-ml Erlenmeyer flask with fresh medium (50 ml) and incubated under the same conditions for 25 d. Mycelia were harvested and washed with distilled water to remove polysaccharides using vacuum filtration. The mycelial pellets were frozen at −40 °C overnight and then vacuum freeze dried using a freeze dryer (VirTis Co., Gardiner, NY) at room temperature for 1 d and stored at −80 °C before processing for DNA extraction. When used for RNA isolation, fresh mycelial pellets were frozen in liquid nitrogen and immediately subjected to extraction.

### DNA isolation and genome sequencing

Freeze-dried mycelia were ground with liquid nitrogen and incubated in CTAB containing 1% β-Mercaptoethanol at 65 °C for 1 h. The supernatant was then extracted with an equal volume of chloroform-isoamyl alcohol (24:1). The extraction was repeated until no more precipitate formed. DNA was precipitated with 2:3 (vol:vol) of cold isopropanol and 1:10 (vol:vol) of 3 M NaAc (pH = 5.2), centrifuged at 10,000 rpm for 15 min at 4 °C, washed twice with 70% cold ethanol and treated with 1 ml of 10 mM Tris-HCl containing 20 μl of 100 mg/ml RNase A for 1 h at 37 °C. After chloroform-isoamyl alcohol extraction, and re-precipitation with cold isopropanol and NaAc at −20 °C for 2 h, DNA was washed twice, first with 70% and then with 100% cold ethanol. Air dried DNA was dissolved in 10 mM Tris-HCl (pH = 8.0). The amount and quality of total DNA was visualized by running out on a 1% agarose gel and quantified with a NanoDrop 1000 Spectrophotometer (Thermo Scientific). A 20 K library was prepared with the total genomic DNA and three SMRT cells were sequenced using PacBio RS II sequencing platform (Pacific Biosciences, Nextomics Biosciences Co., Ltd., Wuhan).

### Assembly of the mitochondrial genome and PCR verification

After removing the adapter sequences, reads with length <50 bp or average quality <0.75 were defined as low-quality and removed. The mitochondrial sequences were extracted from the filtered reads containing both nuclear and mitochondrial genomes, using BLASR^41^ which matches each read against the fungal mitochondrial genome database^25^. The mitochondrial reads were preassembled and corrected using BLASR. The corrected reads were retained and fully assembled with the Celera Assembler program^42^. The assembly was further refined with Quiver^43^. The mt DNA was circularized, resulting in a finished mt circular genome. Average coverage depths were calculated with SAMTools^44^. The mt genome assembly was verified by PCR amplification using seven pairs of primers (Supplementary Table S1) which were designed to target several ambiguous regions and regions with relatively low coverage.

### Mitochondrial genome annotation

Conserved protein-coding and rRNA genes were identified by BLASTn^45^. Intron-exon boundaries of protein- coding and rRNA genes were identified by Clustal W alignment^46^ with intron-less homologous genes from three closely related species, i.e. *Cordyceps militari*s, *C. brongniartii* and *C. bassiana*, combined with locating the start and stop codons. Boundaries of mt small subunit rRNA (*rns*) and intron types (groups I and II and their subgroups) were also checked by RNAweasel^31^. Three programs including tRNAscan-SE^47^, ARAGORN^48^ and RNAweasel were used to predict tRNA genes. RNAweasel identified the most abundant tRNA genes (27) which included all the predictions by tRNAscan-SE (20) and ARAGORN (25). ncORFs in the intergenic and intronic regions longer than 300 bp were predicted using ORF Finder^49^. Predicted ncORFs were analysed by a BLASTx search against the non-redundant protein database in NCBI^50^, using the Mold, Protozoan, and Coelenterate Mitochondrial Code. The mitochondrial genetic map was generated with Circos software^51^ and modified by Adobe Illustrator® CS5 (Version 15.0.0, Adobe®, San Jose, CA). The annotated mt genome of *O. sinensis*, strain 1229, has been submitted to GenBank (Accession number KP835313).

### Identification of repetitive sequences

Repetitive sequences were identified and analysed with different programs. Local BLASTn searches^52^ of mtDNA against itself was performed using a cut-off e-value of 10^−5^. REPuter^53^ was used to identify and locate forward, reverse, complementary and inverted (palindrome) repeats using default settings. Tandem repeats were analyzed by the Tandem Repeats Finder (TRF) program^54^. Simple sequence repeats (SSRs) were detected by the MIcroSAtellite (MISA) identification tool^55^. Short inverted repeats were investigated using EMBOSS software^56^. Dispersed and inverted repeats were visualized by Circos.

### Phylogenetic inference

To evaluate the application of mt genomes for fungal phylogeny, a phylogenetic tree was constructed for the order *Hypocreales* using protein sequences of 14 conserved protein-coding genes found in the mt genome of *O. sinensis* in the present study, including *cox1*, *cox2*, *cox3*, *atp6*, *atp8*, *atp9*, *nad1*, *nad2*, *nad3*, *nad4*, *nad5*, *nad4L* and *nad6*. Accessions of completely sequenced mt genomes of 16 species in *Hypocreales* were retrieved from NCBI Organelle Genome Resources website^25^. In addition, the mt genomes of nine species from other orders of the class *Sordariomycetes* were used as outgroups. The hypocrealean species included in the analyses were *Acremonium chrysogenum* (NC_023268), *A. implicatum* (NC_026534), *Beauveria bassiana* (NC_010652), *B. pseudobassiana* (NC_022708), *Cordyceps bassiana* (NC_017842), *C. brongniartii* (NC_011194), *C. militaris* (NC_022834), *Fusarium circinatum* (NC_022681), *F. gerlachii* (NC_025928), *F. graminearum* (NC_009493), *F. oxysporum* (NC_017930), *F. solani* (NC_016680), *Gibberella moniliformis* (NC_016687), *Hypocrea jecorina* (NC_003388), *Lecanicillium muscarium* (NC_004514), *Metacordyceps chlamydosporia* (NC_022835) and *Metarhizium anisopliae* (NC_008068); and the nine outgroup species were *Ceratocystis cacaofunesta* (NC_020430), *Chaetomium thermophilum* (NC_015893), *Colletotrichum lindemuthianum* (NC_023540), *Madurella mycetomatis* (NC_018359), *Neurospora crassa* (NC_026614), *Podospora anserina* (NC_001329), *Sporothrix schenckii* (NC_015923) and *Verticillium dahliae* (NC_008248). Protein sequences were aligned using the software MAFFT v7.149b^57^ and the alignment was trimmed with trimAl^58^ under a strict model to remove ambiguous regions. Phylogenetic analyses were performed with a maximum likelihood method using RAxML v.7.2.6^59^, assuming the LG substitution matrix and default parameters. Bootstrap values were computed with 100 resampling iterations using an approximate likelihood ratio test.

### RNA preparation, transcriptome sequencing and mitochondrial gene expression analyses

Total RNA was extracted from freshly grown mycelia with TRIzol^®^ Reagent (Life Technologies, Inc., Grand Island, NY) and treated with DNase I (GenStar Biosolutions Co., Ltd., Beijing, China). The quality and concentration of the RNA were assayed in an Agilent 2100 Bioanalyzer and Agilent RNA 6000 Nano kit, respectively. RNA extracts with high purity and quality were selected for cDNA library construction. Oligo (dT) magnetic beads were used for purifying mRNA from total RNA. Fragmentation buffer treated mRNA (200 nt) were used as the templates for cDNA synthesis. A double-stranded cDNA library was constructed with the NEBNext Ultra Directional RNA Library Prep Kit for Illumina and sequenced on the Illumina HiSeq^TM^ 2500 platform at the Nextomics (Wuhan, China). Raw reads were filtered and normalized using NGS QC Toolkit^60^. Adaptor polluted reads and low quality reads (determined as read length of quality score <20 greater than 30%, otherwise determined as high quality) were removed. Filtered high quality reads were mapped to exons of all the conserved protein-coding genes and rRNA genes (*rnl* and *rns*), as well as tRNA genes and ncORFs. RPKM (reads per kilobase exon model per million mapped reads) values^61^ were calculated for all these genes and ncORFs. Genes were considered expressed if RPKM > 0.2.

## Additional Information

**How to cite this article**: Li, Y. *et al.* Complete mitochondrial genome of the medicinal fungus *Ophiocordyceps sinensis*. *Sci. Rep.* **5**, 13892; doi: 10.1038/srep13892 (2015).

## Supplementary Material

Supplementary Figure S1

Supplementary Tables S1-S6
