# Supplementary material for: Complete mitochondrial genome of the medicinal fungus Ophiocordyceps sinensis
Source: Sci Rep. 2015 Sep 15;5:13892. doi: 10.1038/srep13892 (PMC4570212; doi:10.1038/srep13892)
Supplement: Supplementary Figure S1 [file srep13892-s1.doc]

Complete mitochondrial genome of the medicinal fungus *Ophiocordyceps sinensis*

Yi Li^1,2^, Xiao-Di Hu^1,3^, Rui-Heng Yang^1,3^, Tom Hsiang^4^, Ke Wang^1,3^ De-Quan Liang^5^, Fan Liang^5^, De-Ming Cao^5^, Fan Zhou^5^, Ge Wen^5^ & Yi-Jian Yao^1^

^1^State Key Laboratory of Mycology, Institute of Microbiology, Chinese Academy of Sciences, Beijing 100101, China; ^2^College of Plant Protection, Fujian Agriculture and Forestry University, Fuzhou 350002, China; ^3^University of Chinese Academy of Sciences, Beijing 100049, China; ^4^School of Environmental Sciences, University of Guelph, Ontario, N1G 2W1, Canada; ^5^Nextomics Biosciences Co., Ltd., Wuhan 430075, China.

Correspondence and requests for materials should be addressed to Y.-J.Y. (yaoyj@im.ac.cn)

Supplemental figures:


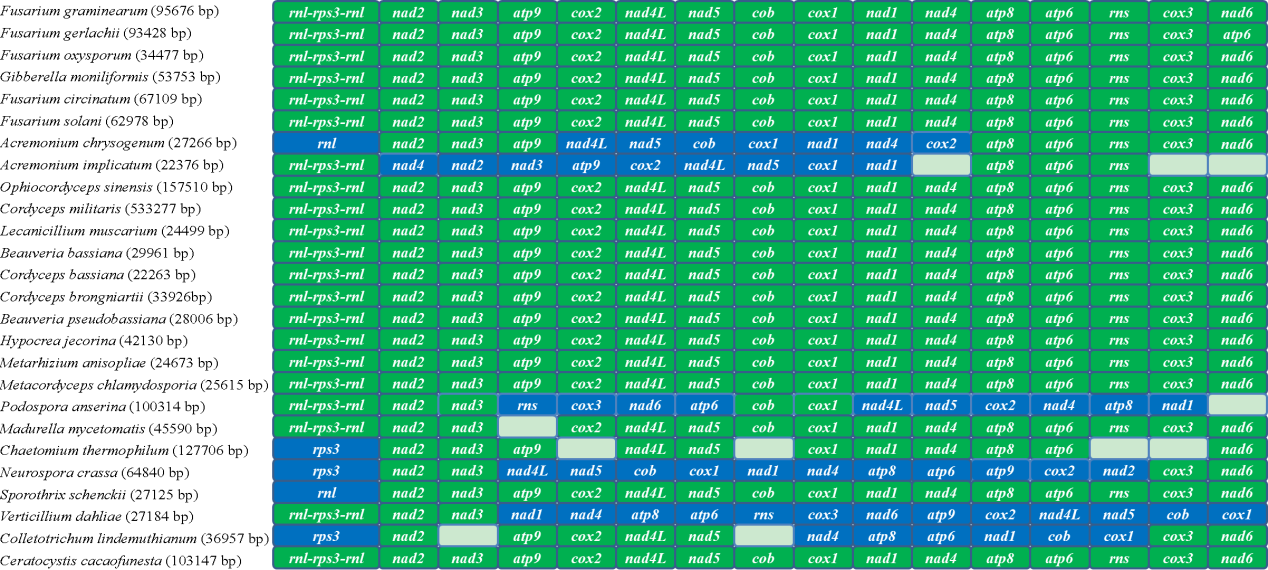


Figure S1: Mitochondrial genome sizes, gene contents and order of species in *Sordariomycetes*. Conserved loci are in green while the variables are in blue. Gene losses are marked as blank boxes. All the genes are aligned and the *rnl* is defined as the initial gene. The *rps3* is usually located within a group I intron of *rnl* and hence is shown together with *rnl* as ‘*rnl-rps3-rnl*’ here.
