# Supplementary material for: Complete mitochondrial genome of the medicinal fungus Ophiocordyceps sinensis
Source: Sci Rep. 2015 Sep 15;5:13892. doi: 10.1038/srep13892 (PMC4570212; doi:10.1038/srep13892)
Supplement: Supplementary Tables S1-S6 [file srep13892-s2.pdf]

Complete mitochondrial genome of the medicinal fungus *Ophiocordyceps sinensis*

Yi Li[1,2], Xiao-Di Hu[1,3], Rui-Heng Yang[1,3], Tom Hsiang[4], Ke Wang[1,3] De-Quan Liang[5], Fan Liang[5], De-Ming Cao[5], Fan Zhou[5], Ge Wen[5] & Yi-Jian Yao[1]

[1]State Key Laboratory of Mycology, Institute of Microbiology, Chinese Academy of Sciences, Beijing 100101, China; [2]College of Plant Protection, Fujian Agriculture and Forestry University, Fuzhou 350002, China; [3]University of Chinese Academy of Sciences, Beijing 100049, China; [4]School of Environmental Sciences, University of Guelph, Ontario, N1G 2W1, Canada; [5]Nextomics Biosciences Co., Ltd., Wuhan 430075, China.

Correspondence and requests for materials should be addressed to Y.-J.Y. (yaoyj@im.ac.cn)

Table S1. Primers used for verification.

Primers	Sequences	Location	Length (bp)	Mismatches detected
OSMT1F	5'-AACATATAGGTGTTACTAGACCAGC-3'	5070–5094	1465	0
OSMT1R	5'-CTCTGAAATACGATAGAGTTTCTTAAC-3'	6507–6534		
OSMT2F	5'-AAGTTAAGGGTGGAGATCCGTC-3'	22247–22268	1736	0
OSMT2R	5'-GCTTCAATATTTCACTCTACCAATTGAG-3'	23954–23981		
OSMT7F	5'-ATAGCTATGACTTTTAAGAGTTCG-3'	139643–139666	1257	1 indel
OSMT7R	5'-TGTATGCCAGCTAATATCTTATCATCG-3'	140874–140900		
OSMT6F	5'-TATTAGCCCTATCGTAAAAGGTAAAAGC-3'	142509–142536	1849	0
OSMT6R	5'-TTGTACCGCGAGAGTGTGGTTAC-3'	144335–144357		
OSMT5F	5'-GGTACCATACTAGTACTAAATAACACTAG-3'	144263–144291	1639	2 indels
OSMT5R	5'-ACCTGCTTTATCCCTTATGCGCTGTG-3'	145871–145901		
OSMT4F	5'-CGATATAAGAACAAGTGAATTACAAAGC-3'	149268–149250	1639	1 indel
OSMT4R	5'-GTGCTTCCATATGTGTATACATAG-3'	150883–150906		
OSMT3F	5'-GTAGGCTTATGAATAATTAATTCATCG-3'	154175–154201	1608	1 indel
OSMT3R	5'-TAAGCTGTAGCTAGGTTACTCAAAGC-3'	155757–155782		
Total			11193	5

Table S2. Annotated conserved protein-coding and rRNA genes in the mt genome of *Ophiocordyceps sinensis* .

Gene	Start	End	Strand	Length (bp)	Introns			Exon length (bp)	Coding sequence density
					group I	group II	uncertain		
rnl	1	19704	+	19704	6	2	1	3629	18.42%
rps3	14837	16418	+	1582				1582	100.00%
nad2	29905	36732	+	6828	2	1		1731	25.35%
nad3	36733	37146	+	414				414	100.00%
atp9	37382	38676	+	1295	1			225	17.37%
cox2	38810	50132	+	11323	5			705	6.23%
nad4L	50367	52143	+	1777	1			270	15.19%
nad5	52143	62698	+	10556	5			1957	18.54%
cob	64355	79753	+	15399	5	1		1161	7.54%
cox1	80393	111166	+	30774	12	1		1581	5.14%
nad1	111930	123377	+	11448	3			1164	10.17%
nad4	123878	125209	+	1332				1332	100.00%
atp8	127206	127352	+	147				147	100.00%
atp6	127438	133685	+	6248	2		1	777	12.44%
rns	135851	139570	+	3720		1		1694	45.54%
cox3	142695	146413	+	3719	2			810	21.78%
nad6	148250	149694	+	1445			1	668	46.23%
Total					44	6	2		

Table S3. Transfer RNA genes in the mt genome of *Ophiocordyceps sinensis*.

tRNA	Codon usage	Start	End	Length (bp)	Strand
tRNA-Ala	GCA	25665	25737	73	+
tRNA-Arg	CGU	50208	50280	73	+
	AGA	111311	111383	73	+
tRNA-Asn	AAC	142566	142639	74	+
tRNA-Asp	GAC	139705	139777	73	+
tRNA-Cys	UGC	79904	79975	72	+
tRNA-Gln	CAA	28237	28310	74	+
tRNA-Glu	GAA	20009	20081	73	+
tRNA-Gly	GGA	26398	26468	71	+
	GGA	148100	148170	71	+
tRNA-His	CAC	28500	28574	75	+
tRNA-Ile	AUA	146749	146820	72	+
	AUC	151682	151753	72	+
tRNA-Leu	UUA	20231	20313	83	+
	CUA	27781	27864	84	+
tRNA-Lys	AAA	24181	24253	73	+
tRNA-Met	AUG	20085	20155	71	+
	AUG	20158	20230	73	+
	AUG	29556	29626	71	+
tRNA-Phe	UUC	23944	24016	73	+
tRNA-Pro	CCA	157361	157433	73	+
tRNA-Ser	AGC	140645	140726	82	+
	UCA	155644	155729	86	+
tRNA-Thr	ACA	19707	19778	72	+
tRNA-Trp	UGA	155837	155908	72	+
tRNA-Tyr	UAC	139599	139682	84	+
tRNA-Val	GUA	150983	151055	73	+
Total				2016	

Table S4. Lengths and types of all the introns in the mt genome of *Ophiocordyceps sinensis* .

Gene	Intron 1	Intron 2	Intron 3	Intron 4	Intron 5	Intron 6	Intron 7	Intron 8	Intron 9	Intron 10	Intron 11	Intron 12	Intron 13	Total length (bp)	Average length (bp)
cox1	2535/IB	1504/IB	2843/IB	3267/IB	1165/IB	2242/ID	1054/IB	1124/IB	1663/IB	1262/IB	2478/II	6435/IB	1616/IB	29188	2245
rnl	2938/II	1439/IA	1243/IA	1358/IC2	522	1343/IC2	2091/II	3869/IA	1272/IA					16075	1786
cob	2525/II	2588/IB	3228/IC2	1726/IB	2039/IA	2132/IA								14238	2373
cox2	1412/IC2	1527/IC2	2504/IC2	3232/IC2	1943IC1									10618	2124
nad5	1958/ID	2968/ID	1181/ID	1108/IB	1384/IC2									8599	1720
nad1	5139/IB	3634/IA	1511/IB											10284	3248
nad2	1396/IC2	2338/II	1363/IC2											5097	1699
atp6	1533/IB	3855/IC2	83											5471	1824
cox3	1411/IB	1498/IC2												2909	1455
rns	2026/II													2026	2026
nad4L	1507/IC1													1507	1507
nad6	777													777	777
atp9	1070/IA													1070	1070
Total														107859	2004

Underlined, derived group I introns.

Table S5. Non-conserved open reading frames (ncORFs) in the mt genome of *Ophiocordyceps sinensis* .

ncORFs	Reading frame	Start	End	Length	Location	Annotation	E-value	Accession
ncORF1	1	2338	2895	558	rnl-i1/II	reverse transcriptases (RTs) with group II intron origin	2.46E-25	cd01651
ncORF2	-3	2412	2717	306	rnl-i1/II	reverse transcriptases (RTs) with group II intron origin	1.24E-20	cd01651
ncORF3	1	4408	4764	357	rnl-i2/I	GIY-YIG endonuclease	1.62E-23	cd10445
ncORF4	-2	4795	5142	348	rnl-i2/I	GIY-YIG endonuclease	0.00003	YP_009072317.1
ncORF5	1	6878	7180	303	rnl-i4/I	hypothetical protein		
ncORF6	1	9515	9829	315	rnl-i6/I	LAGLIDADG endonuclease	5.95E-15	pfam00961
ncORF7	1	9866	10432	567	rnl-i6/I	LAGLIDADG endonuclease	6.06E-10	pfam00961
ncORF8	3	12322	12714	393	rnl-i7/I	LAGLIDADG endonuclease	1.15E-08	pfam00961
ncORF9	1	18287	18880	594	rnl-i9/I	LAGLIDADG endonuclease	6.73E-18	pfam03161
ncORF10	-2	30812	31123	312	nad2-i1/I	LAGLIDADG endonuclease	0.00816	pfam00961
ncORF11	1	32528	32842	315	nad2-i2/II	reverse transcriptases (RTs) with group II intron origin	1.95E-20	cd01651
ncORF12	1	32897	33277	381	nad2-i2/II	reverse transcriptases (RTs) with group II intron origin	6.34E-16	cd01651
ncORF13	1	33590	34108	519	nad2-i2/II	reverse transcriptases (RTs) with group II intron origin	6.86E-11	pfam01348
ncORF14	2	35112	35567	456	nad2-i3/I	LAGLIDADG endonuclease	6.84E-18	pfam00961
ncORF15	1	39014	39361	348	cox2-i1/I	LAGLIDADG endonuclease	4E-20	YP_008757687.1
ncORF16	2	40833	41345	513	cox2-i2/I	LAGLIDADG endonuclease	3.8E-18	pfam00961
ncORF17	3	42886	43212	327	cox2-i3/I	LAGLIDADG endonuclease	1.61E-11	pfam00961
ncORF18	1	43550	44257	708	cox2-i4/I	GIY-YIG endonuclease	2.17E-19	cd10445
ncORF19	2	45423	45854	432	cox2-i5/I	GIY-YIG endonuclease	0.000000007	CDL73257.1
ncORF20	2	48993	49724	732	cox2-i6/I	GIY-YIG endonuclease	2E-144	AGN49031.1
ncORF21	2	51267	51701	435	nad4L-i/I	LAGLIDADG endonuclease	2.83E-14	pfam00961
ncORF22	2	52710	53060	351	nad5-i1/I	LAGLIDADG endonuclease	1.75E-08	pfam00961
ncORF23	2	53214	53534	321	nad5-i1/I	LAGLIDADG endonuclease	1E-39	YP_009072396.1
ncORF24	1	58367	58708	342	nad5-i3/I	hypothetical protein		
ncORF25	1	61445	61840	396	nad5-i5/I	LAGLIDADG endonuclease	1.53E-13	pfam00961
ncORF26	3	62818	63321	504	intergenic region	fibronectin-attachment protein	0.0000055	pfam07174
ncORF27	-3	63328	63744	417	intergenic region	SDA1 protein	0.000557	pfam05285
ncORF28	3	64903	65238	336	cob-i1/II	Reverse transcriptases	3E-14	YP_008964951.1
ncORF29	3	65566	66399	834	cob-i1/II	Reverse transcriptases (RTs) with group II intron origin	4.75E-23	cd01651
ncORF30	-1	65769	66209	441	cob-i1/II	Reverse transcriptases (RTs) with group II intron origin	1.01E-13	cd01651
ncORF31	-1	67371	67682	312	cob-i2/I	LAGLIDADG endonuclease	0.000362	pfam00961
ncORF32	2	70827	71978	1152	cob-i3/I	hypothetical protein		
ncORF33	3	73993	74835	843	cob-i4/I	LAGLIDADG endonuclease	0.00087	pfam00961
ncORF34	3	75022	75477	456	cob-i5/I	reverse gyrase	0.00322	PRK14701
ncORF35	1	75764	76369	606	cob-i6/I	LAGLIDADG endonuclease	9.38E-29	pfam03161

ncORF36	1	82148	82651	504	cox1-i1/I	GIY-YIG endonuclease	0.0000032	cd10445
ncORF37	-3	88090	88458	369	cox1-i4/I	LAGLIDADG endonuclease	0.000000564	pfam00961
ncORF38	1	88187	89179	993	cox1-i4/I	LAGLIDADG endonuclease	3.56E-15	pfam00961
ncORF39	2	100143	100511	369	cox1-i10/II	hypothetical protein		
ncORF40	1	101168	101686	519	cox1-i10/II	Reverse transcriptases (RTs) with group II intron origin	1.35E-09	cd01651
ncORF41	3	101965	102486	522	cox1-i10/II	Reverse transcriptases (RTs) with group II intron origin	0.000794	pfam01348
ncORF42	3	104119	104706	588	cox1-i11/I	LAGLIDADG endonuclease	5.83E-12	pfam00961
ncORF43	2	105141	105554	414	cox1-i11/I	hypothetical protein		
ncORF44	2	105693	106247	555	cox1-i11/I	LAGLIDADG endonuclease	1E-56	CDL73453.1
ncORF45	3	106360	106671	312	cox1-i11/I	hypothetical protein		
ncORF46	1	115202	115651	450	nad1-i1/I	GIY-YIG endonuclease	2E-29	YP_007507077.1
ncORF47	3	122482	122880	399	nad1-i3/I	GIY-YIG endonuclease	1.37E-10	cd00283
ncORF48	3	125650	126075	426	intergenic region	hypothetical protein		
ncORF49	2	130923	131291	369	atp6-i2/I	GIY-YIG endonuclease	2.48E-11	cd00283
ncORF50	3	131599	131940	342	atp6-i2/I	GIY-YIG endonuclease	2E-28	YP_001427393.1
ncORF51	2	132342	133292	951	atp6-i2/I	GIY-YIG endonuclease	6.39E-10	cd10445
ncORF52	1	133703	134245	543	intergenic region	hypothetical protein		
ncORF53	1	134279	134821	543	intergenic region	hypothetical protein		
ncORF54	1	141638	142102	465	intergenic region	hypothetical protein		
ncORF55	3	144532	144867	336	cox3-i2/I	LAGLIDADG endonuclease	5E-25	YP_001249345.1
ncORF56	-3	153193	153591	399	intergenic region	DNA-dependent RNA polymerase	1.03E-11	COG5108
ncORF57	-3	153682	154128	447	intergenic region	T3/T7-like RNA polymerase	1.18E-12	PHA00452
ncORF58	-3	154501	155490	990	intergenic region	hypothetical protein		
				28335				

Table S6. Transcription analyses of conserved protein-coding genes and ncORFs.

Gene	Exon length	Mapped reads	RPKM	ncORFs	Length	Mapped reads	RPKM
atp6	777	126	3.630905634	ncORF1	558	85782	3455.421157
atp8	147	5	0.764524996	ncORF2	306	1	0.073454362
atp9	225	252	25.17427906	ncORF3	357	128	8.058992897
cob	1161	1076	17.16486127	ncORF4	348	328	21.18525126
cox1	1581	6888	51.76255976	ncORF5	303	557	41.31916972
cox2	705	361	11.86287952	ncORF6	315	55	3.924561645
cox3	812	757	20.95457562	ncORF7	567	234	9.276236616
nad1	1164	586	11.31575811	ncORF8	393	4202	240.3269734
nad2	1731	159	2.064614989	ncORF9	594	1322	50.02464664
nad3	414	435	23.61717433	ncORF10	312	207	14.91264814
nad4	1332	179	3.020562495	ncORF11	315	37	2.640159652
nad4L	270	110	9.157310505	ncORF12	381	117	6.902396537
nad5	1957	699	8.028332845	ncORF13	519	117	5.067077226
nad6	668	163	5.484665696	ncORF14	456	5	0.246458716
rnl	3629	75209	465.8240055	ncORF15	348	150	9.688377102
rns	1696	74467	986.9088185	ncORF16	513	129	5.652119881
rps3	1582	3127	44.42837425	ncORF17	327	7	0.481159768
tRNA-Ala(TGC)	73	6	1.847427524	ncORF18	708	143	4.539853089
tRNA-Arg(TCT)	73	2	0.615809175	ncORF19	432	124	6.451741492
tRNA-Asp(GTC)	73	9	2.771141286	ncORF20	732	46	1.412491263
tRNA-Gln(TTG)	74	3	0.911231144	ncORF21	435	117	6.045547312
tRNA-Glu(TTC)	73	18	5.542282572	ncORF22	351	45	2.881671138
tRNA-Gly(TCC)	71	10	3.16577956	ncORF23	321	189	13.23414203
tRNA-His(GTG)	75	64	19.18040309	ncORF24	342	252	16.5620257
tRNA-Ile(GAT)	72	7	2.18526728	ncORF25	396	465	26.39348792
tRNA-Ile(TAT)	72	56	17.48213824	ncORF26	504	4435	197.7889875
tRNA-Leu(TAA)	83	8	2.166461193	ncORF27	417	11455	617.4446871

tRNA-Leu(TAG)	84	18	4.816507473	ncORF28	336	2182	145.9669348
tRNA-Lys(TTT)	73	27	8.313423858	ncORF29	834	1877	50.58680391
tRNA-Met(CAT)	71	10	3.16577956	ncORF30	441	0	0
tRNA-Phe(GAA)	73	15	4.61856881	ncORF31	312	10	0.720417784
tRNA-Pro(TGG)	73	15	4.61856881	ncORF32	1152	1221	23.82331561
tRNA-Ser(GCT)	82	3	0.822330544	ncORF33	843	8	0.213305195
tRNA-Ser(TGA)	86	6	1.568165224	ncORF34	456	1	0.049291743
tRNA-Thr(TGT)	72	21	6.555801839	ncORF35	606	24	0.890179599
tRNA-Trp(TCA)	72	2	0.62436208	ncORF36	504	285	12.71022806
tRNA-Tyr(GTA)	84	8	2.140669988	ncORF37	369	84	5.116723386
tRNA-Val(TAC)	73	2	0.615809175	ncORF38	993	133	3.010519273
		164909		ncORF39	369	1563	95.20760301
				ncORF40	519	4402	190.6433671
				ncORF41	522	896	38.58127059
				ncORF42	588	52	1.987764989
				ncORF43	414	370	20.08817127
				ncORF44	555	68	2.753943012
				ncORF45	312	322	23.19745266
				ncORF46	450	78	3.896019379
				ncORF47	399	2819	158.8039131
				ncORF48	426	574	30.28595779
				ncORF49	369	238	14.49738293
				ncORF50	342	1768	116.1970692
				ncORF51	951	890	21.03529026
				ncORF52	543	102	4.222205446
				ncORF53	543	6136	253.9946335
				ncORF54	465	61	2.948600274
				ncORF55	336	150	10.03439057
				ncORF56	399	1	0.056333421

			ncORF57	447	2	0.100568389
			ncORF58	990	102	2.315815715
					137068	
